# Performance of IRS on malaria prevalence and incidence using pirimiphos-methyl in the context of pyrethroid resistance in Koulikoro region, Mali

**DOI:** 10.1186/s12936-020-03357-8

**Published:** 2020-08-12

**Authors:** Fousseyni Kané, Moussa Keïta, Boïssé Traoré, Sory Ibrahim Diawara, Sidy Bane, Souleymane Diarra, Nafomon Sogoba, Seydou Doumbia

**Affiliations:** Malaria Research and Training Centre; International Center for Excellence in Research (ICER-Mali); Faculty of Medicine and Odonto Stomatology, University of Sciences, Techniques and Technologies of Bamako (USTTB), Bamako, Mali

**Keywords:** Parasitaemia, Incidence, Malaria, IRS, LLINs, Mali

## Abstract

**Background:**

Koulikoro Health District is one of three districts of Mali where the indoor residual spray (IRS) has been implemented from 2008 to 2016. With widespread of resistance to pyrethroid, IRS was shifted from pyrethroid to pirimiphos-methyl from 2014 to 2016. The objective of this study was to assess the added value of IRS to LLINs on the prevalence of parasitaemia and malaria incidence among children under 10 years old.

**Methods:**

A comparative study was carried out to assess the effects of pirimiphos-methyl based IRS on malaria prevalence and incidence among children from 6 months to 10 years old in selected pyrethroid resistance villages of two health districts in Mali: one where IRS was implemented in combination with LLINs (intervention area) and one with LLINs-only (control area). Two cross-sectional surveys were carried out at the beginning (June) and end of the rainy season (October) to assess seasonal changes in malaria parasitaemia by microscopy. A passive detection case (PCD) was set-up in each study village for 9 months to estimate the incidence of malaria using RDT.

**Results:**

There was an increase of 220% in malaria prevalence from June to October in the control area (14% to 42%) versus only 53% in the IRS area (9.2% to 13.2%). Thus, the proportional rise in malaria prevalence from the dry to the rainy season in 2016 was 4-times greater in the control area compared to the IRS area. The overall malaria incidence rate was 2.7 per 100 person-months in the IRS area compared with 6.8 per 100 person-month in the control areas. The Log-rank test of Kaplan–Meier survival analysis showed that children living in IRS area remain much longer free from malaria (Hazard ratio (HR) = 0.45, CI 95% 0.37–0.54) than children of the control area (P < 0.0001).

**Conclusions:**

IRS using pirimiphos-methyl has been successful in reducing substantially both the prevalence and the incidence of malaria in children under 10 years old in the area of pyrethroid resistance of Koulikoro, Mali. Pirimiphos-methyl is a better alternative than pyrethroids for IRS in areas with widespread of pyrethroid resistance.

## Background

During the past 15 years, substantial financial investment has been made in the fight against malaria worldwide. Indeed, funding for control and elimination increased by about US$ 60 million between 2010 and 2015 [1]. These investments increased the different control strategies implemented by the different National Malaria Control Programmes (NMCP) (Prevention, Diagnosis, Treatment, and Surveillance) to achieve the objectives of the Global Technical Strategy 2016–2030 for malaria control [2]. As a result, of those efforts, substantial reduction in the number of malaria cases worldwide (from 237 million in 2010 to 216 million in 2016) was reported. However, sub-Saharan Africa still accounts for 90% of the malaria burden at the global level [3, 4].

Access to prevention is an essential component in the fight against malaria. Current prevention strategies are based on the correct and early management of malaria cases, chemoprevention by intermittent preventive treatment (IPT) in pregnant women and seasonal malaria chemoprevention (SMC) in children from 3 to 59 months, the use of long-lasting insecticidal nets (LLINs) and indoor residual spraying (IRS) [2, 5, 6]. Each of the last two strategies has shown significant results in reducing the burden of malaria in Africa [4, 7, 8]. Studies have also demonstrated the public health impact and cost-effectiveness of the combination of LLINs and IRS [9, 10]. However, the rapid spread of vector resistance to insecticide is a challenge for malaria control programmes.

In Mali, the presence and spread of the *kdr* mutation, which encodes for pyrethroids and DDT resistance has been documented [11–14]. After introduction of IRS in 2008, because of the vector resistance to lambda-cyhalothrin deltamethrin, Mali shifted from pyrethroid to a carbamate in 2011, and then to organophosphate (pirimiphos-methyl) in 2014. This study aimed to assess effects of a pirimiphos-based IRS by comparing malaria prevalence and incidence in children from 6 months to 10 years old in selected pyrethroid resistance villages of Koulikoro district, where IRS and LLINs were used in combination (IRS area), and its neighbor district of Banamba, where only LLINs were used (control area). The terms “IRS area” and “control area” will be used to represent respectively IRS + LLINs and LLINs-only through the document.

## Methods

### Study design and sites selection

Cross-sectional and passive case detection surveys were undertaken in two health districts to assess the effects of pirimiphos-methyl based IRS in the context of vector resistance to pyrethroids, on *Plasmodium falciparum* malaria parasitaemia and incidence in children from 6 months to 10 years old.

**Fig. 1:**
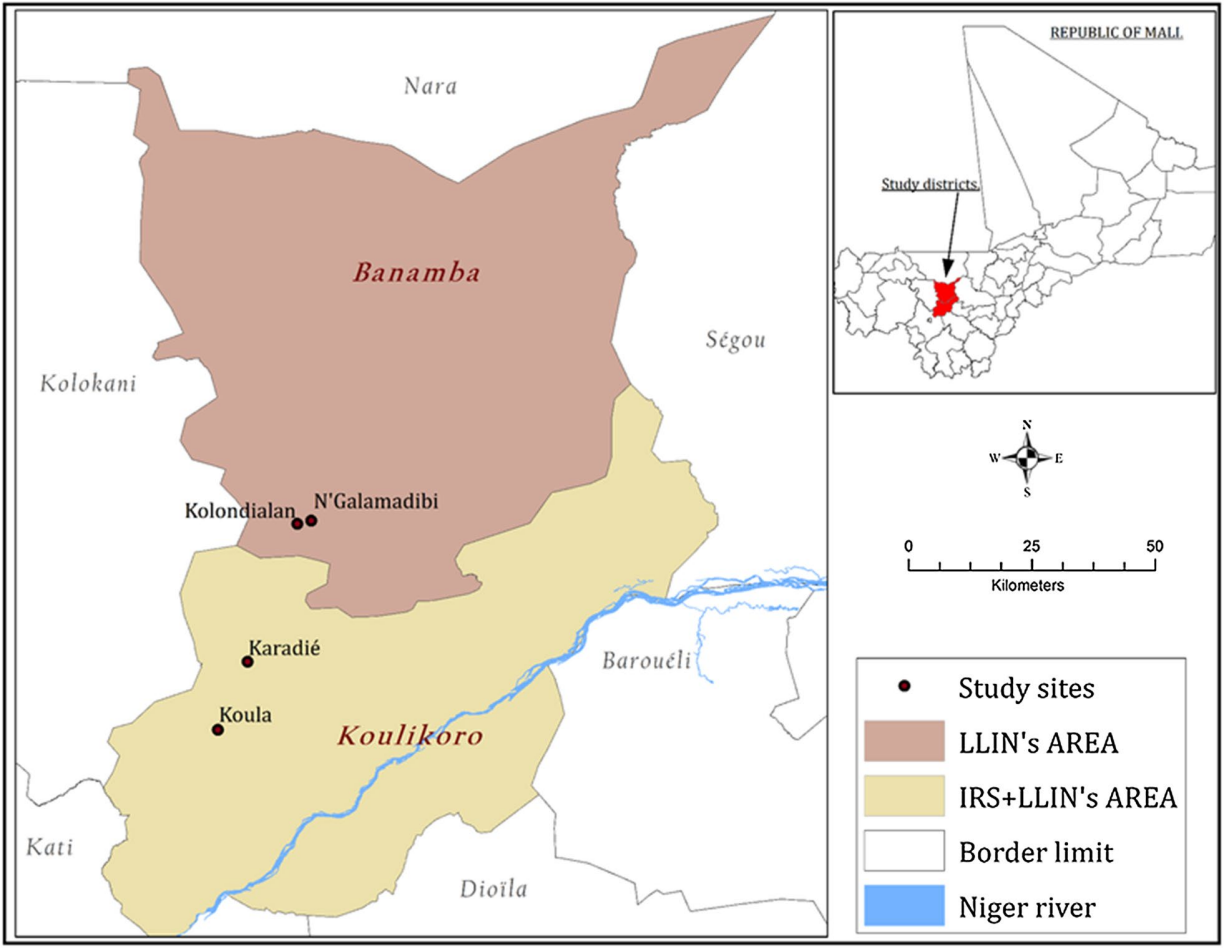
Map of the districts of Koulikoro and Banamba showing the selected villages

The study sites were selected after performing a World Health Organization standard bioassay test [15] in many villages of Koulikoro and Banamba health districts to determine malaria vector resistance status to pyrethroids. Koula (7.65 W, 13.12 N) and Karadié (7.60 W, 13.24 N) in Koulikoro health district, and Kolondialan (7.51 W, 13.49 N) and N’Galamadibi (7.48 W, 13.48 N) in Banamba health district (Fig. 1) presenting comparable pyrethroid resistance status were then selected to represent the areas with IRS and control area, respectively. Table 1 shows the characteristics of the different selected sites. In both areas, the mean annual rainfall fluctuates between 600 and 1200 mm. The monthly mean temperature during the rainy season varies between 29 and 33 °C. *Anopheles gambiae* sensu lato (*s.l*.) is the major malaria vector (> 98%) in all the villages and malaria control core intervention are mainly LLINs, IRS, and SMC. Malaria transmission occurs mostly during the rainy season (June to October) with a mean monthly mosquito man-biting rate reaching its peak in August/September.

**Table 1:** Characteristics of the selected study sites in the health districts of Koulikoro and Banamba

Villages	Districts	Total population	Population of 0–10 years	Phenotypic resistance	Resistance mechanisms	Control intervention	Ecological zone
Koula	Koulikoro	9003	3476	6 (100)	KdrW (29.6%)	LLINs, IRS, SMC	Sudano-Sahelian
Karadié	Koulikoro	4854	1923	29 (100)	KdrW (49.0%)	LLINs, IRS, SMC	Sudano-Sahelian
N’Galamadibi	Banamba	5074	2008	29 (100)	KdrW (48.0%)	LLINs, SMC	Sudano-Sahelian
Kolondialan	Banamba	4311	1687	24.5 (150)	KdrW (49.0%)	LLINs, SMC	Sudano-Sahelian

### Study population and screening

A population census was carried out in the selected villages by the research team. A unique identifier was assigned to children from 6 months to 10 years. Eligible children were enrolled after their parents or legal guardians agreed to comply with study procedures in a written informed consent. Since 2014, LLINs coverage was scaled up to universal (two people for one net) through mass distribution campaigns. For the purpose of our study, in collaboration with the NMCP, new LLINs were distributed to all participants in both areas just after the first cross-sectional survey in June 2016. The pirimiphos-methyl based IRS was implemented in July 2016 in the IRS area with a coverage rate of 97.1%.

### Cross-sectional surveys

Data on the malaria prevalence study were collected through two cross-sectional surveys: in June (at the onset of the rainy season) and in October (at the end of the rainy season). In June 2016, a total of 950 children 6 months to 10 years old were enrolled in the IRS area (620 in Koula and 330 in Karadié) and 621 in the control areas (230 in N’Galamadibi and 391 in Kolondialan). In October 2016, 915 volunteers were enrolled in the IRS area (601 in Koula and 314 in Karadié) and 594 in the control areas (218 in N’Galamadibi and 376 in Kolondialan). During each survey, demographic and clinical data, haemoglobin levels, and blood smears were collected from each participant. Parents/guardians of all children were interviewed on the ownership and use of LLINs the previous night before the survey. Any participant with body temperature ≥ 37.5 °C was considered as suspected case of malaria, tested with rapid diagnostic test (RDT). RDT positives cases were treated for free by the local health staff according to the national malaria control policy.

### Passive case detection (PCD)

Data on malaria incidence was collected through a passive case detection set up on each study site in collaboration with the local health staff. Study participants with fever were invited at the health center (suspected malaria cases) and tested with RDT. RDT positive cases were treated for free according to the national policy by the local health staff.

### Microscopy

The thick and thin blood films collected during the cross-sectional surveys were stained with 10% Giemsa and examined under the 100× oil immersion objective lens of a light microscope. The number of asexual and sexual parasites was counted against 200 leucocytes.

### Data analysis

Data was collected on case report forms (CRFs) and entered into to Microsoft Excel v. 2016. The analysis was performed in R-studio 1.1.41 [16] and GraphPad Prism v.7 Software for Windows[17].

From the cross-sectional survey data, the prevalence of malaria parasitaemia was defined as the proportion of subjects with microscopy *P. falciparum* positive smear, and the gametocyte rate the proportion of those who were carrying gametocytes based on microscopy. Malaria clinical case was defined as fever (axillary temperature ≥ 37.5 °C, as measured by a standard electronic thermometer) and RDT positive. The prevalence of anaemia was defined as the proportion of children with haemoglobin levels < 8 g/dl. The LLINs usage rate was the proportion of participants/guardians who reported that they slept under a net the previous night before the survey. From the PCD data, the malaria incidence rate was estimated as the number of new malaria cases (positive RDT) per person-months during the 9 months follow-up period (expressed per 100 person-months).

The Pearson χ^2^ test was used to compare the proportions and the Student’s t test to compare the averages between IRS and control sites. The associations between the risk factors and the parasitaemia were assessed using univariate and multivariate logistic forward stepwise regression models. The Kaplan–Meier survival analysis was used to compare the average duration between malaria cases in the two study areas. The log-rank test was used to compare malaria risk between the IRS and control areas over the 9 months follow-up period (July 2016 to March 2017).

## Results

### Cross-sectional survey

A total of 1571 children were enrolled at the first survey (May–June 2016) and 1509 in the second survey (October 2016), with 4% lost to follow-up during the second passage. The mean age of the study population was 5.6 ± 2.8 years and the sex ratio 1.1 for the male. As shown in Table 2, in June representing the start of the rainy season, malaria parasitaemia was significantly lower (P = 0.0027) in the IRS area (9.2%, n = 950) compared to the control area (14.0%, n = 621). For the gametocyte index, there was not a significant difference between the two areas. Surprisingly prevalence of fever cases was higher (P = 0.0058) in the IRS area (12.8%, n = 122) than in the control area (8.4%, n = 52). LLINs usage was higher (P < 0.001) in the IRS area (91.7%, n = 871) compared to the control area (20.9%, n = 130). However, a significant increase in the utilization of the LLINs in the control area was observed, rising from 20.9% in June to 89.2% in October 2016. More anaemia cases (P < 0.001) were observed in the control area (53.0%, n = 323) than in the IRS area (32.2%, n = 306).

**Table 2:** Malaria parasite rate, reported fever, LLINs usage and anemia prevalence in children of 6 months to 10 years in both areas in June and October 2016

Malaria indices	June 2016			October 2016		
	IRS area (N = 950) Freq (%)	Control area (N = 621) Freq (%)	χ^2^ P-value	IRS area (N = 950) Freq (%)	Control area (N = 621) Freq (%)	χ^2^ P-value
Parasitemia	87 (9.15)	87 (14.0)	0.0027*	121 (13.22)	253 (42.25)	0.0027
Gametocyte index	24 (2.53)	14 (2.25)	0.7316	29 (3.17)	68 (11.45)	< 0.0001
Fever	122 (12.84)	52 (8.37)	0.0058	196 (21.42)	275 (53.70)	< 0.0001
LLINs	871 (91.68)	130 (20.93)	< 0.0001	886 (96.83)	530 (89.23)	< 0.0001
Anemia	306 (32.21)	329 (52.97)	< 0.0001	409 (44.70)	364 (61.28)	< 0.0001

In October, representing the end of the rainy season and the peak of transmission, malaria parasitaemia remained significantly lower (P < 0.001) in the IRS area (13.2%, n = 950) compared to the control (42.3%, n = 621). However, in both areas, there was an increase in the parasite rate from June to October. This increase was 53.0% (9.2% vs 13.2%) in the IRS area, and up to 220.0% (14.0% *vs* 42.3%) in the control area. In contrast to June, where there was no significant difference between the two areas in the gametocyte index which was significantly higher (P < 0.001) in the control area (11.5%) compared to the IRS area (3.2%) in October. The same pattern was observed with the fever. LLNs usage increased in both areas in October compared to June. However, it remained lower in the control area (89.2%, n = 530) compared to the IRS area (96.8%, n = 886).

**Fig. 2:**
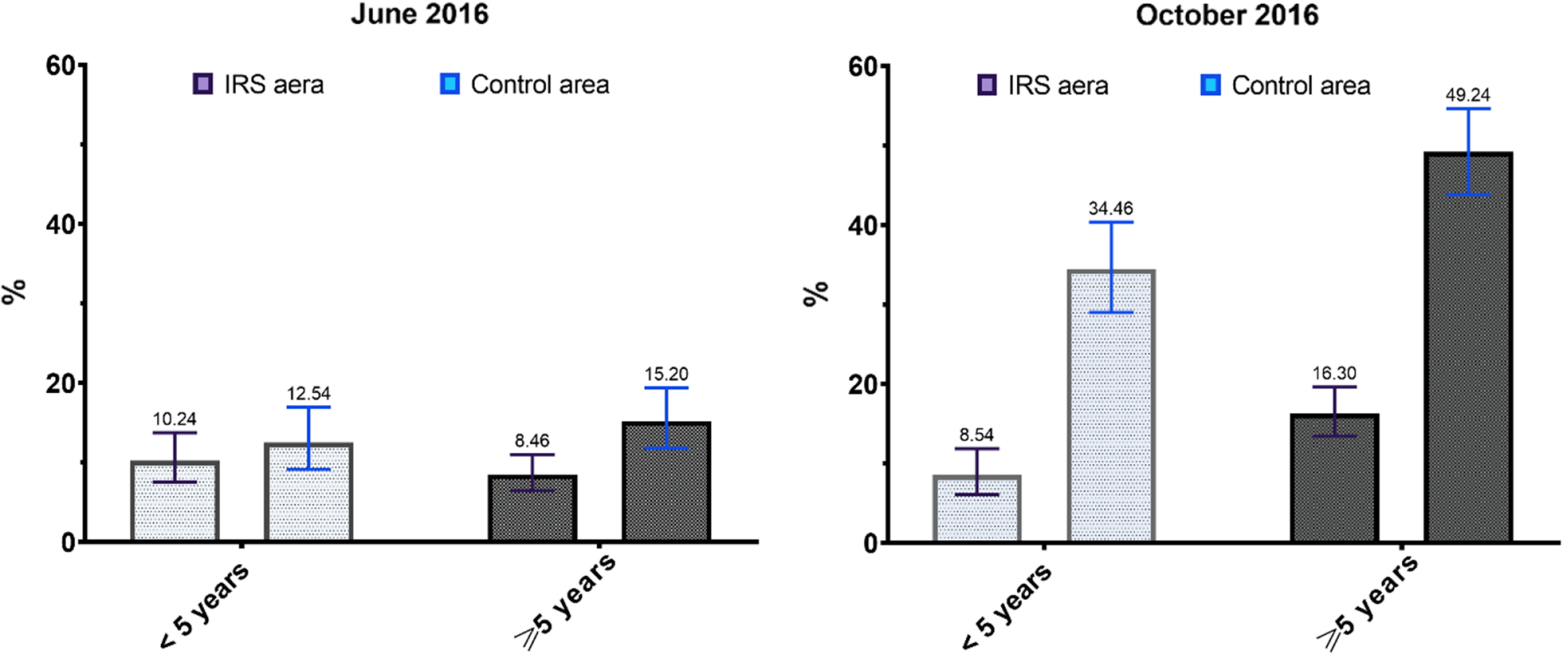
Malaria parasitemia in children of 6 months to 10 years by age group in areas of IRS and control in June and October 2016

Figure 2 shows the variation of the asexual parasite rate by age group in the two areas. In June there was no significant difference in parasite rate between the two age groups of the same area as well as between the two areas. In October, the parasite rate was significantly higher (P = 0.0007; P = 0.0003) in children of 5–10 years than in those < 5 years in the same area as well as in between areas (P < 0.0001).

Table 3 presents the results of the univariate logistic regression analysis performed on the data of the cross-sectional survey in October 2016 in the two study areas. Children were more likely to be infected with malaria parasites in the control area compared to the IRS area (OR = 4.2, 95% CI 3.7–6.2), and, in the older children (> 5 years) compared to the younger (OR = 1.6, 95% CI 1.3–2.1). Having fever, anaemia, and antecedent of fever in the previous 2 weeks were all positively associated with parasite carriage (Table 3). Children owing LLINs were not more f protected (OR = 0.6, 95% CI 0.8–0.01; P = 0.0503) than those who did not.

**Table 3:** Simple logistic regression between parasitemia and area, age group, fever and LLINs usage, anemia among children of 0–10 years old during the peak of transmission (October 2016)

	Parasitemia	
	Crude OR (95% CI)	*P* value
Areas		
IRS area	1	
Control area	4.9 (3.7–6.2)	< 0.001***
LLINs		
No		
Yes	0.6 (0.8–1.01)	0.05031
Age		
< 5 years	1	
≥ 5 years	1.6 (1.3–2.1)	< 0.001***
Fever		
No	1	
Yes	4.6 (3.3–6.4)	< 0.001***
Anemia		
No	1	
Yes	1.4 (1.1–1.8)	0.002**
Fever since week		
No	1	
Yes	2 (1.6–2.6)	< 0.001***

**Fig. 3:**
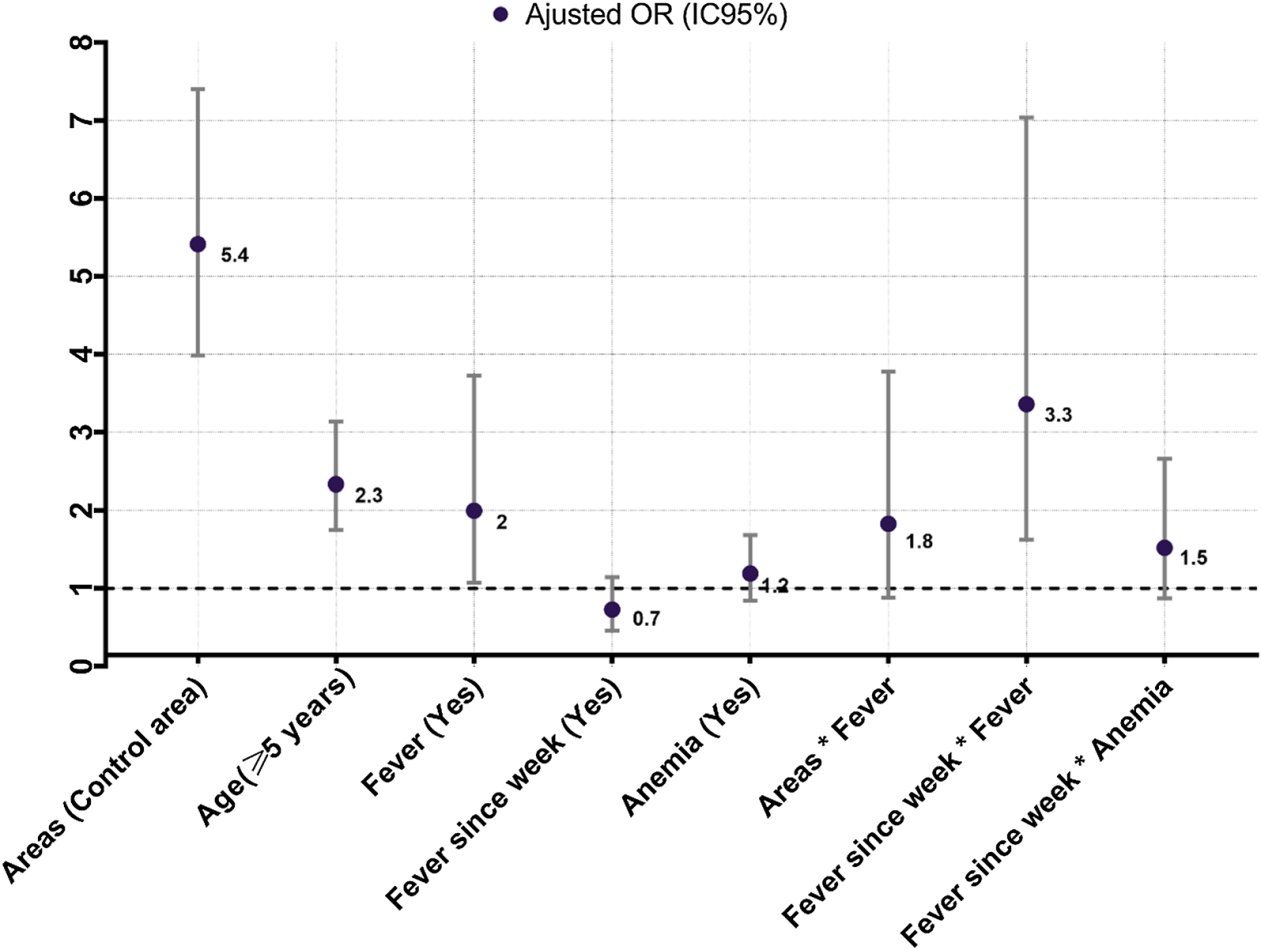
Results of the stepwise multivariate logistic regression model between independent explanatory variables and parasitemia during October 2016 survey in the two study areas

Figure 3 shows results of the final stepwise multivariate regression model. Interactions between explanatory variables were assessed by including proper cross-product terms in the regression models. The likelihood ratio test was used to compare model with and without the inter-action term to estimate the significance of the interaction. The likelihood ratio test was used to compare model with and without the inter-action terms to estimate the significance of the interactions. The LLINs was excluded from the final model, which showed an increase in the risk of parasitaemia in the control area (OR adjusted = 5.6, P < 0.0001). Children > 5 years old were twice more likely (OR adjusted = 2.3, P < 0.0001) to carry parasite compared to < 5 years old. The risk of infection was significantly associated with fever (OR adjusted = 2) compared to no fever cases (P = 0.028). There was no significant association between anaemia and history of fever.

### Passive case detection

**Fig. 4:**
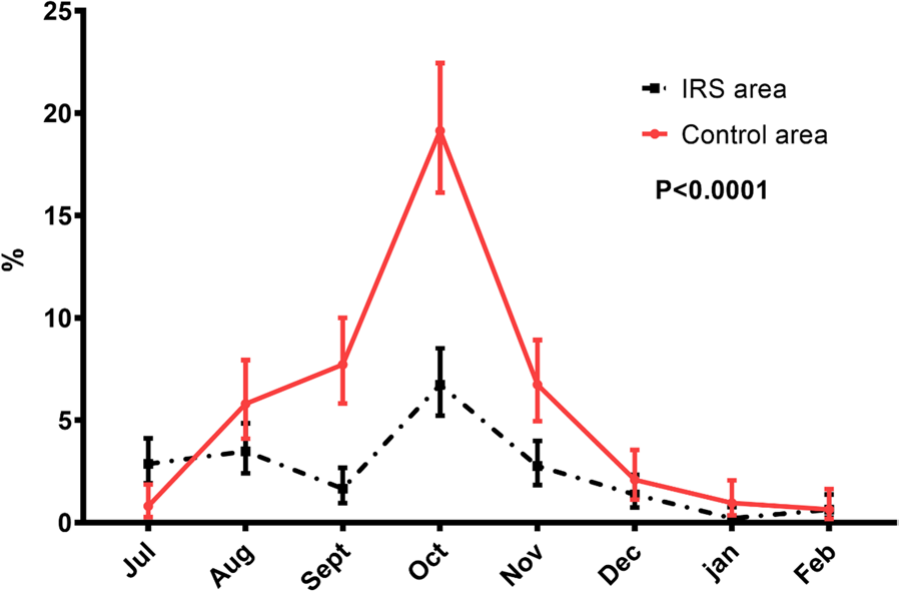
Monthly incidence of malaria (TDR+, Temperature > 37 °C) in children of 6 months to 10 years old in the IRS areas (continue line) and control (dash line) from July 2016 to February 2017

The overall incidence rate in children of 6 months–10 years was 2.7 for 100 person-month in the IRS area and 6.8 for 100 person-month in the control area. However, during the first 2 months (July–August) and after the end of the rainy season (December–February), there was not a significant difference in malaria incidence between the two areas as shown in Fig. 4.

It was from September to November that malaria incidence was significantly lower in the IRS area compared to the control area. As in malaria parasitaemia, the peak of malaria incidence was observed in October in both areas but was much lower in the IRS area than the control area.

**Fig. 5:**
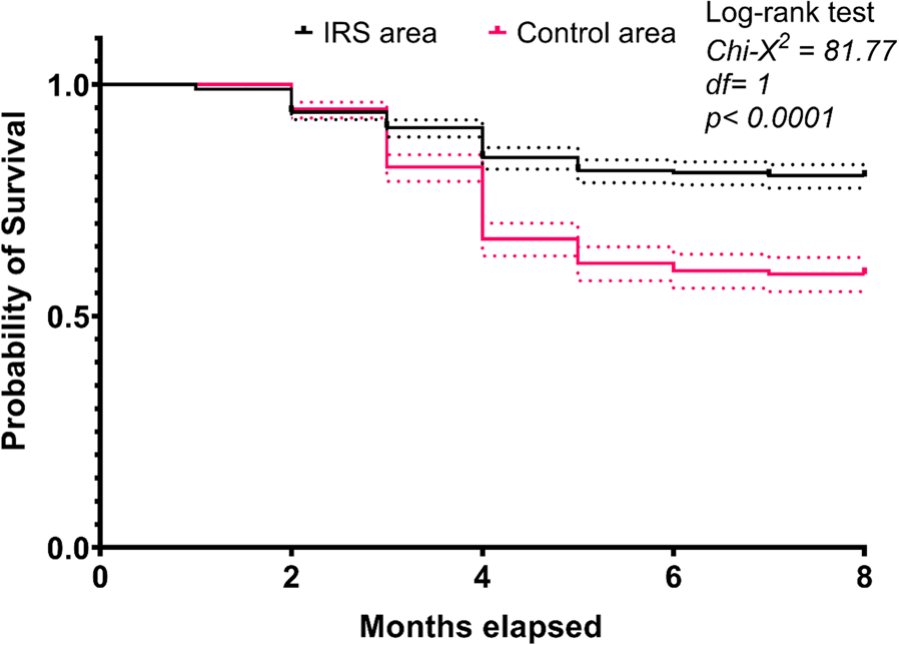
Kaplan–Meier survival curve of children of 1–10 years old living in the study areas after 8 months of follow-up

Using the 9-months follow-up data of the study participants, the average duration of the clinical malaria in the two areas was estimated using the Kaplan–Meier survival analysis. The survival curve showed that children living in IRS area were more likely to be free of malaria longer compared with the control area (Fig. 5). The log-rank test showed that this difference was significant (Log-rank test Chi- χ2 = 81.77, df = 1, P < 0.0001).

## Discussion

In this study, malaria prevalence and incidence in two areas of integrated malaria control strategies were compared to evaluate the effect of the added value of IRS. Data were collected in both areas through two cross-sectional surveys and 9 months of passive case detection in the health clinics. The results of the cross-sectional surveys at both the start and end of malaria transmission season showed that malaria parasitaemia was significantly lower in the IRS area (LLINs + IRS) compared to the control area (with LLINs-only). Since the two areas are comparable in terms of malaria epidemiology and interventions, this difference can be attributed to the added value of the IRS in the IRS area. Indeed, it is of common knowledge that each of the LLINs [9, 18, 19] and IRS [20–22] significantly reduce malaria burden when deployed separately. Thus, their integration is expected and supported by many studies [7, 21, 23–25] to make more reduction in malaria burden compared to their respective single impact. Despite the IRS, there was an increase in malaria parasitaemia from the start to the end of the rainy season as expected in seasonal malaria transmission areas where transmission intensity rich it peak at the end of the rainy season[26]. However, the increase in the IRS area was much lower (53.0%) than in the control area (220.0%). The nine consecutive years of IRS campaign have certainly contributed to reduce and even to suppress the peak of the transmission in the IRS area. Also, there was not a difference in gametocyte index between the two areas at the start. At the end of the rainy season, this index was significantly higher in the control area (11.5%) compared to the IRS area (3.2%). This observation partially explains the lowest prevalence of malaria parasitaemia in the IRS area because gametocyte index is the potential source of infection for mosquito, hence malaria transmission [26, 27].

The results of the logistic regression analysis showed the actual risk related to malaria infection in the control area, and highlighted that association between fever and parasitaemia was overestimated, and that anaemia was a confounding factor.

Regardless of the study area, malaria parasitaemia was higher in the age group of 5–10 years than in the < 5 years (Table 3). This unexpected observation was also reported by Touré et al., who reported that children of 6–9 years old were at least twice more likely to carry parasites than children < 5 years old [28]. Explanation to this observation was the expansion and focus of current control interventions (LLINs, SMC, IPT) on children < 5 years. Walldorf et al. also reported that school-age children and adults less exposed to anti-malarial interventions were representing a reservoir of malaria infection in replacement of children < 5 years [29].

As with the parasitaemia, malaria incidence in children of 0–10 years old was significantly lower in the IRS area compared to the control area. The traditional transmission peak was observed in both areas, but it was much lower in the IRS area than in the control area (Fig. 3). Indeed, while the insecticide can last in LLINs over about 3 years, for IRS it last over a maximum of 6 months. Thus, the IRS campaign is applied once a year with the objective to cover the peak of the transmission. As shown on the Kaplan–Meier survival curve (Fig. 4), children living in the IRS area were less likely to develop malaria clinical case than in the control area. This is in line with others studies in which significant reduction in malaria incidence was reported when scaling-up IRS in combination with other interventions [30]. However, the protection period of the residual effects of IRS was limited in the time suggesting that this strategy may need to be improved by using longer lasting insecticides or by increasing its application frequency per year [31].

The difference observed in the prevalence of anaemia between the IRS and control areas, between the age groups and the participant with and without fever, was a good indirect indicator of the strategies used [32–34].

## Conclusions

This study have shown that pirimiphos-methyl based IRS is successful in reducing substantially the prevalence and the incidence of malaria in children under 10 years old in area of pyrethroids resistance of Koulikoro and children living in the IRS area remained much longer free from malaria than those of the control area. The study suggests that pirimiphos-methyl is a suitable insecticide for malaria control in areas with widespread of pyrethroid resistance.

## Data Availability

The datasets generated during and/or analysed during the current study are available from the corresponding author on reasonable request.

## Abbreviations

IRS: Indoor Residual Spraying
LLINs: Long-lasting Insecticidal Nets
IPT: Intermittent Preventive Treatment
SMC: Seasonal Malaria Chemoprevention
MOH: The Ministry of Health
MRTC: Malaria Research and Training Center
NMCP: The National Malaria Control Programme
IPT: Preventive Intermittent Therapy
CRFs: Case Report Forms
RDT: Rapid Diagnostic Test
USTTB: University of Sciences, Techniques and Technologies of Bamako
